# Who's the Boss? Assessing Convergent Validity of Aggression Based Dominance Measures in Male Laboratory Mice, *Mus Musculus*

**DOI:** 10.3389/fvets.2021.695948

**Published:** 2021-07-09

**Authors:** Amanda J. Barabas, Jeffrey R. Lucas, Marisa A. Erasmus, Heng-Wei Cheng, Brianna N. Gaskill

**Affiliations:** ^1^Department of Animal Science, Purdue University, West Lafayette, IN, United States; ^2^Department of Biological Sciences, Purdue University, West Lafayette, IN, United States; ^3^United States Department of Agriculture, Agricultural Research Service, Livestock Behavior Research Unit, Purdue University, West Lafayette, IN, United States

**Keywords:** aggression, darcin, dominance, home cage behavior, mus musculus, preputial gland, social network analysis, tube test

## Abstract

Aggression among group housed male mice continues to challenge laboratory animal researchers because mitigation strategies are generally applied at the cage level without a good understanding of how it affects the dominance hierarchy. Aggression within a group is typically displayed by the dominant mouse targeting lower ranking subordinates; thus, the strategies for preventing aggression may be more successful if applied specifically to the dominant mouse. Unfortunately, dominance rank is often not assessed because of time intensive observations or tests. Several correlates of dominance status have been identified, but none have been directly compared to home cage behavior in standard housing. This study assessed the convergent validity of three dominance correlates (urinary darcin, tube test score, preputial gland to body length ratio) with wound severity and rankings based on home cage behavior, using factor analysis. Discriminant validity with open field measures was assessed to determine if tube test scores are independent of anxiety. Cages were equally split between SJL and albino C57BL/6 strains and group sizes of 3 or 5 (*N* = 24). Home cage behavior was observed during the first week, and dominance measures were recorded over the second. After controlling for strain and group size, darcin and preputial ratio had strong loadings on the same factor, which was a significant predictor of home cage ranking showing strong convergent validity. Tube test scores were not significantly impacted by open field data, showing discriminant validity. Social network analysis revealed that despotic power structures were prevalent, aggressors were typically more active and rested away from cage mates, and the amount of social investigation and aggression performed by an individual were highly correlated. Data from this study show that darcin and preputial ratio are representative of home cage aggression and provide further insight into individual behavior patterns in group housed male mice.

## Introduction

Excessive aggression in male mice is a leading welfare problem in the animal laboratory which can impact data validity and numbers of animals used in experiments. Many solutions offered to mitigate excessive aggression have been proposed, but inconsistencies occur between studies (1, 2). This may not be surprising because most aggression studies only measure behavior at the cage level, not at the individual level. According to (3), the term dominance is used to indicate the outcome between individuals during competition over resource or during a negative interaction, with a reasonable degree of predictability. While dominance is most often associated with aggression, in primates dominance status is in fact best predicted by the number of retreats performed by a subordinate, regardless of whether an aggressive act preceded it (3, 4). In mice, dominance and aggression can be one and the same, as the mouse who attacks most also receives the most overall submissions (5, 6). In general, aggression is only one component of dominance, but it is the behavior of concern in a vivarium. Thus, individual ranking should be considered when trying to reduce aggression in the home cage. In order to evaluate ranking and the hierarchy in the cage, valid measures of dominance are necessary. This will help researchers understand the motivations behind excessive aggression.

Past behavioral analyses show that male mice form complex social hierarchies, with most groups displaying a linear or despotic power structure (5–12). However, in depth behavioral observations, like those done with social network analysis, are time intensive, making them impractical for quick evaluation. A dominance measure that requires less time to quantify, and one that can be validated based on relationships developed within the cage, would be a more realistic option. While less time intensive measures of dominance exist, they have only been compared to behavior in resident-intruder tests or complex group competitions and may not reflect behavior in a typical laboratory cage (2).

One commonly used measure of dominance is the tube test (13, 14). In brief, pairwise trials are conducted between cage mates in an arena composed of two Plexiglas chambers connected by a PVC tube. Contestants are placed at each end of the tube, locomote to the center, and the less dominant one will back out upon encountering the opponent. The tube test is meant to replicate competitive situations without exposing the mice to direct conflict. As reviewed by (15), stable tube test scores correlate with agonistic behavior, urine marking, and resource possession; however, there may also be a learning element involved, requiring mice to undergo repeated trials for stable results (16, 17). Data from (18) supports a learned component to tube test outcomes, where mice kept in long-term familiar groups displayed considerable rank variation over three trials, suggesting that scores are affected by the duration of the test and the test environment. Indeed, less than half of male mice competing in the tube test maintained a consistent ranking over three trials, and many groups displayed a dynamic, unstable relationship (19). Currently, no studies have compared time dependent tube test ranks to other dominance correlates. These types of comparisons can assess convergent validity [how well-similar measures reflect the same construct (20)] and discriminant validity [dissimilarity between measures that reflect different constructs (20)] of dominance correlates, to identify which measures accurately portray home cage interactions.

In addition to exploring the convergent validity of tube test scores with other dominance measures, this study aims to compare tube test scores with measures of anxiety from the open field maze (OFM). This question arose from past work, where tube test ranking did not predict levels of two urinary pheromones that are known to differ between dominant and subordinate mice (21). Therefore, it is possible that the tube test may reflect other behavior that is not necessarily associated with dominance *per se*, such as anxiety or perhaps general locomotor activity. The tube test is conducted outside of the home cage, and it is possible that anxiety may cause mice to remain in the tube out of thigmotactic comfort, and not dominance over an opponent. Further, models of chronic social defeat have been shown to be related to higher levels of anxiety in various assays (22, 23). Thus, it is possible that victimized mice remain in the tube out of security. Recently, a systematic review showed that measures of anxiety are not significantly different between dominant and subordinate mice (24). However, the high level of study heterogeneity found in this review could mask an effect from social rank. The tube test was only used in 35% of included studies, so a direct link between those scores and anxiety could have been lost (24). Additionally, the tube test could be subjected to effects of general activity: it is possible that a mouse could win simply by being inactive and waiting for an opponent to retreat (25). Past work has shown that general activity measured in the OFM does not relate to tube test rank, but mice competed in daily tube test trials for a week and likely became familiar with the expectations in that arena (26). It is unknown if locomotion plays a role for mice who may not be as familiar with the tube test arena. Assessing discriminant validity should help provide an answer to whether measures from the tube test are associated with anxiety or locomotion.

While behavioral tests can be beneficial, other measures may be more accurate at indicating dominance in the home cage as they do not require an external testing arena and therefore are not subjected to the same confounding environmental factors. One such indicator of aggression is the Pelt Aggression Lesion Scale (PALS). This method evaluates wound severity and is a validated indicator of wounding, specifically due to aggression (27). However, it is unknown how PALS relates to individual behavior and has only been used to assess substantial wounding in black mice who have pigment follicles that burst with injury. Another measure of dominance is the ratio of preputial gland weight to body length, which increases in males with less wounding and in those who display more attack behavior (28, 29). While potentially useful, mice used in previous studies were housed in isolation between weaning and the study period, calling into question the social competency of these test subjects. Even if socially competent, it is unknown whether this pattern holds true for mice housed in stable groups. Further, some research found conflicting evidence of this association where no relationship was found between the preputial glands and social status. However, these analyses were based solely on gland weight, not the relation to body size (30, 31). A final physiological measure is urinary levels of MUP20 (darcin) which has been connected to social rank, with mice who display more attack behavior, win more conflicts, and possess a more desirable nest site producing higher levels than opponents or other enclosure occupants (32–34). Again, these studies were not done in stable groups of mice but were either based on resident-intruder trials or complex competition arenas where distinct territories could be formed.

Additionally, there is much to learn about how dominance measures and aggression may relate to other behaviors within the home cage. Anecdotal observations from our lab have shown that mice who attack most often also sleep away from cage mates and build separate nests, which aligns with historical observations of wild *Mus musculus* (35). To our knowledge, the only formal assessment of the relationship between resting location and aggression found that mice who attack more spend more time resting away from cage mates (5). While the (5) study provides support for our own observations, the sample period from their study only consisted of 2 h per day and may have missed occurrences of other mice resting on their own. Another behavior that might provide insight into the social dynamic within the cage is allo-grooming, which has rarely been studied. Previous research suggests that allo-grooming is most often performed between subordinate mice (5); however, more recent work found that an individual's place in a grooming network does not relate to their place in aggression networks (9). Other behaviors of interest may be specific only to subordinate mice. Various primate species respond submissively to those above them in rank and this pattern extends to various mouse strains (3, 5, 6). However, several strains of inbred mice are known for excessive inter-male aggression [i.e., SJL (36)], and it is worth exploring if persistent fighting is due to a lack of appropriate submissive behavior by low ranking mice. Contrasting claims also exist regarding social investigation behavior (i.e., sniffing). It is often used as a measure of sociability toward stranger mice and has been considered a neutral exploratory behavior (37–39), but recent work has shown it to be predictive of aggression (9) and at the group level, it correlates with aggressive behavior (21).

This study aimed to assess the convergent validity of three dominance measures (tube test score, preputial gland to body length ratio, and urinary darcin) with PALS score and home cage dominance ranking based on an aggression focused social network analysis (SNA). We considered measures to have strong convergent validity if they loaded strongly on the same axis of a factor analysis and were significant predictors of home cage dominance in a linear model. This study also aimed to test the discriminant validity of tube test scores with two measures of anxiety and one of locomotion in a novel environment. This would be indicated by a lack of significance in a linear mixed model. Additionally, this study sought to address four aims focused on home cage interactions in an aggression focused SNA: (1) assess how strain and group size may influence power distribution of male mice housed in standard shoebox cages; (2) examine how individual attack behavior relates to socio-positive behaviors and time spent in proximity to other cage mates; (3) determine if victim mice respond appropriately to aggression; and (4) conduct a formal analysis on how, social investigation behavior correlates with submissive and aggressive behaviors.

## Methods

### Ethics Statement

All animal use was approved by Purdue University's Institutional Animal Care and Use Committee under protocol #1707001598 (not previously submitted as a Registered Report).

Due to concern over excessive home cage aggression, humane endpoint criteria required any mouse with wounding >1cm^2^ to be immediately euthanized. Animals were monitored daily for wounding, general activity, and signs of pain/distress. Four cages reached our criteria during the study (see Supplementary Table 1 for more information).

### Animals

This study used a 2 × 2 factorial design based on strain and group size. *A-priori* sample size was determined using Mead's Rule (40). In total, 48 SJL/JOrlcoCrl (SJL) and 48 B6N-Tyr^c−Brd^/BrdCrCrl (albino B6) specific pathogen-free mice were acquired from Charles River (Wilmington, MA) and housed in groups of three or five, *N* = 24 cages. Albino B6 were chosen over pigmented B6 in order to ensure researchers and care staff could not distinguish strains based on coat color. Five is a common group size in a typical shoebox cage but less aggression has been observed in groups of three (41, 42). Treatments were replicated in time with three batches of cages each time, due to spatial constraints. Each batch contained *n* = 2 cages per strain x group size combination. Mice arrived at ~8 weeks of age and were housed for 2 weeks in open top micro-isolator cages (Ancare, Bellmore, NY) with customized lids (Alternative Design, Siloam Springs, AR) and external water bottles for overhead viewing (Supplementary Figure 1). Food (Envigo, Teklad 2016, Indianapolis, IN) and reverse osmosis water were offered *ad libitum*. Cages contained aspen wood chip bedding (NEPCO, Warrensburg, NY) and 8.5 g of virgin kraft crinkle paper (Enviro-Dri, Cleveland, Ohio) for nesting material. Cages were kept under a 12:12 light: dark cycle (lights on at 06:00) with relative humidity ranging 24–64% and temperature ranging 17.8–23.3C. Cages were changed weekly, with the exception of two cages (one albino B6 group of 5 and one SJL group of 5) in batch one that were changed on study day 4 and 5, respectively, due to excessive condensation on the cage walls and lid.

A numerical sequence from RANDOM.org was initially used to place cages on a rack shelf. Strain and group size treatments were ultimately balanced across rack shelves and the relative distance to the room's door. Two cages occupied each shelf and were surrounded by white foam board (Office Depot, Boca Raton, FL) as done previously to block background movement during video recordings (21). Each cage was given its own letter label from A to X representing its group size and strain. Only these labels were visible in order to blind caregivers and research staff to strain treatment during sample collection, behavior tests, and video coding. It was only possible to be blind to group size when analyzing data from individual mice.

In the following sections, procedures are listed in the order in which mice experienced them.

### Home Cage Behavior

All mice were individually marked with a fur marker (Stoelting, Wood Dale, IL) and continuously monitored with overhead and side view infrared closed circuit television (CCTV) cameras (Sony, Tokyo, Japan; HDview, Los Angeles, CA) and GeoVision monitoring software (Taipei, Taiwan). Data were analyzed on days 2 and 7 of the study period to capture early interactions during acclimation to the new cage and interactions at the end of the week, when mice were more familiar with each other (21, 43). Each 24-h period, from the 2 days, was watched using all occurrence sampling for 1 min every 5 min. Individual occurrences of the following interaction types were recorded: escalated aggression, mediated aggression, submissive behavior, allo-grooming, and social investigation (Table 1). For each interaction, both the actor and recipient mouse were recorded as well as the time stamp. In the morning before each observation period, individual markings were retraced using permanent marker (Sharpie, Oak Brook, IL) as the fur marker was not visible under infrared lighting.

**Table 1:** Ethogram of observed behavior categories.

**Social Behaviors- actor and recipient recorded every 5 min using all occurrence sampling; the mouse who performed a submissive behavior first was considered the loser of each interaction**		
**Category**	**Behavior**	**Description**
Mediated Aggression	Resource Theft	A mouse will approach another that is either eating a piece of food or chewing on a piece of bedding. The approaching mouse will then attempt to take the resource from the other's paws or mouth. It may or may not be successful. It is often preceded by facial sniffing and involves one or both mice tugging at the resource.
	Tail Rattling	Fast waving movements of the tail. This behavior may be partially obscured by bedding material, but can be detected by displacement of bedding near a mouse's tail.
	Thrust	The aggressor mouse will first threaten its target cage mate by thrusting its head and fore body toward its cage mate's head or body. The aggressor's paw may come in brief contact with the target, but otherwise no contact is made.
	Mounting	Attempts to mount another animal in the absence of intromission. Palpitations with forepaws and pelvic thrusts may be present.
	Chase	A mouse will chase a fleeing partner, but no biting occurs
Escalated Aggression	Bite	The aggressor mouse attacks the recipient with open mouth and appears to bite the recipient, or latches onto the recipient by his teeth. The recipient responds by jumping or fleeing quickly. Aggressor mouse may rush or leap at the victim. This includes any rough and tumble actions and any mouse using its teeth to grab and tug on another's tail. Only score for the mouse that is biting.
	Fighting	Displayed by two or more animals when locked together. Separate behaviors are difficult to distinguish properly due to the fast rolling over and over seen with the animals kicking, biting, and wrestling. The initial victim retaliates toward the attacker. Score for all mice actively involved in the fight.
Submissive	Submissive Upright	A posture where the animal will sit on its haunches in an upright position exposing the belly. The forepaws are off the ground and the mouse may stretch out its forepaws toward the threatening mouse. Mouse can also be laying on its side with one forepaw and one hind paw stretched toward the threatening mouse and its back touching the ground.
	Fleeing	This behavior is characterized by a mouse moving away from the mouse performing an aggressive or investigative behavior. It can also be done by a mouse when it is approached by another. Typically fleeing animals will run, but in a confined space may walk or turn first. Also score if the mouse turns away without locomoting.
Allo-groom	During grooming, the actor mouths and licks the fur on the recipient' body. The actor will also use its teeth to clean the hair shaft by pulling the fur from the base of the hair shaft upward or outward.	
Social investigation	Sniffing directed toward another mouse (face, ano-genital, or body trunk). Only score this behavior if the actor's nose is seen directly oriented at or is close to touching another mouse. This will typically involve a slight head bob. Only score if the sniff lasts at least 1 s.	
**Time Budget- recorded every 5 min using instantaneous scans**		
Active	Score if the mouse is alert and conscious. This includes locomoting around cage, eating/drinking, interacting with cage mates, self-grooming, sniffing the cage/air, or passively sitting in the cage.	
Group Sleep	Sleeping that occurs when two or more mice are resting while in contact with the body of another mouse. When in the nest, the animals may not be seen clearly due to camera angles. If there is no movement in the nest, it is assumed the animals are sleeping. This will typically be in the main nest, but if no nest exists, they could remain behind the same pile of bedding.	
Solitary Sleep	Score if the mouse is seen resting in a location away from a central rest area	

*Descriptions were taken from mousebehavior.org*.

On day 2 and 7, time budget and location data were also recorded for each mouse using instantaneous scan sampling every 5 min. The following behaviors were included in the time budget: active, group sleep, and solitary sleep (Table 1). From these data, we calculated the proportion of observations each mouse spent performing each behavior. For the location data, a 4 × 2 transparent grid was overlaid on the video screen and the square where each mouse was observed was recorded to assess whether mice were alone or together. When active, mice were recorded in the square that contained their head; when resting, mice were recorded in the square that contained more than half of their body. However, when mice were observed resting in a central nest site and that site spanned multiple squares, all mice were documented in the square containing the center of the nest. Location data were used to determine the proportion of observations where mice were observed alone. For all behavior observations, inter-rater reliability was assessed with Cohen's Kappa coefficient based on previous criteria (44). Social behavior and time budget reliability were acceptable at 0.71 and 0.76, respectively. Location reliability was excellent at 0.93. A maximum of two observers coded each behavior category (A.J.B. and a trained undergraduate assistant). Two 24-h periods were used for reliability, equating ~5% of the total video. The first period was randomly selected from the cages of five SJL mice, as it was assumed that they would contain the most aggression. The second period was randomly chosen from cages of three albino B6 to counterbalance strain and group size. While two 24-periods were used for the official reliability calculation, the total amount of training video varied across each student coder. The student who coded location data reviewed ~8% of the entire dataset as this was relatively simple data to record (the mice's location was limited in this housing). The students who coded the time budget and interactions, respectively, each reviewed ~13% of the dataset between practice and reliability.

**Note**: While observing video from day 7 in the first batch of mice (6 cages), individual identities could not be seen in infrared lighting due to inadequate markings. Video data from this time period were omitted from all analyses.

### Urinary Darcin

On study day 7, all mice were individually placed in empty cage bottoms with a wire floor grid to collect fresh urine. Only 70% of mice urinated while on the wire grids. For those that did not produce urine, sample collection was attempted in the OFM or while acclimating to the tube test arena (see methods below). In total, urine was collected from 85% of mice in this study (90% of SJL-5; 87% SJL-3; 76% albino B6-5; 92% albino B6-3). After collection, urine was stored in a −80C freezer until analysis at the Purdue Proteomics Facility (West Lafayette, IN).

Sample preparation followed previous methods (45, 46). Briefly, proteins were precipitated using 4 × the sample volume of acetone and denatured with 40 μL of 8M urea. Bicinchoninic acid assay was used to calculate total protein amount in each sample. 50 μg protein (equivalent volume) was reduced using 10 mM dithiothreitol at 37°C for 1 h followed by alkylation using alkylating reagent (195 uL acetonitrile, 1 μL triethylphosphine and 4 μL of Iodoethanol) and incubated for 1 h at 37°C. After reduction and alkylation, samples were dried in a vacuum centrifuge. The trypsin/LysC mix was dissolved in 400 μL of 50 mM ammonium bicarbonate, and 80 μL was added to each sample for digestion. Digestion was performed at high pressure using a Barocycler (50°C; 60 cycles: 50 s at 20 kPSI and 10 s at 1 ATM). Digested peptides were desalted using MicroSpin columns (C18 silica; The Nest Group), and dried in a vacuum concentrator at room temperature. Dried clean peptides were resuspended in 97% purified water, 3% ACN, and 0.1% FA at a final concentration of 1 μg/μL.

Samples were analyzed by reverse-phase LC-ESI-MS/MS system using the Dionex UltiMate 3,000 RSLC nano System coupled to the Q-Exactive High Field Hybrid Quadrupole Orbitrap Spectrometer (Thermo Fisher Scientific, Waltham, MA) as previously described (45). Peptides were loaded onto a trap column (300 μm ID × 5 mm) packed with 5 μm 100Å PepMap C18 medium, and then separated on a reverse phase column (50-cm long × 75 μm ID) packed with 2 μm 100Å PepMap C18 silica (Thermo Fisher Scientific, Waltham, MA) at a flow rate of 200 nL/min. The column temperature was maintained at 50°C. The positive ion mode was used for all the MS measurements, with 120 min LC gradient and standard data-dependent mode 50. MS data were acquired with a Top 20 data-dependent MS/MS scan method. Instrument calibration was done using calibration mix solution (Thermo Fisher Scientific, Waltham, MA) at the start of each batch run and then after every 72 h. Instrument performance was also evaluated routinely using Hele cell digest (Thermo Fisher).

LC-MS/MS data were analyzed using MaxQuant software (version 1.6.3.3) against the UniProtKB *Mus musculus* genome (85,159 sequences as of Feb. 2020, www.unitprot.org). Default settings were used unless otherwise stated. The following parameters edits were made for this search: 10 ppm precursor mass tolerance; trypsin/Lys-C enzyme specificity; variable modification was oxidation of methionine (M); fixed modification was iodoethanol of cysteine (C); false discovery rate (FDR) of 0.02; peptide spectral match (PSM) and protein identification was set to 0.01. Label free quantitation (LFQ) was selected. All quantifications were calculated by MaxQuant. After the search, peptides with MS/MS counts under two were removed from the dataset. Standardized LFQ values for MUP20/darcin were used for subsequent analyses.

### Open Field Maze

Open field maze (OFM) procedures were based on previous methods (47). Briefly, mice were tested individually in one of two 60 × 60 cm OFM arenas on study day 8. Arenas were cleaned with ethanol and allowed to air dry before the first and between subsequent trials. Mice were handled using plastic tubes (3 7/8“ long x 2” inside diameter; 1/8" wall; BioServ, Flemington, NJ) as traditional tail handling can alter anxiety measures (48). Due to time constraints, half the mice were randomly assigned to morning (07:00–09:00) or afternoon (15:00–17:00) testing, balanced across treatments. All trials were 10 min long and recorded with CCTV cameras (Sony, Tokyo, Japan) for analysis using Ethovision software (Noldus, Wageningen, Netherlands). Grid squares (10 cm^2^) were superimposed over the test arena, and the total distance traveled in cm and percent of time spent outside of the outer edge were calculated. The number of fecal boli were also tallied on testing day.

### Tube Tests

Like in OFM methods, half of the mice were acclimated and tested in the morning and half in the afternoon. Mice kept their same testing time assignment throughout the study. Briefly, the arena consisted of two plexiglass holding areas (approx. 19 cm × 19 cm × 21.5 cm) connected by a PVC tube (approx. 2.5 cm inner diameter). On study day 9, mice were individually acclimated to the arena. Each mouse was given at least 5 min to comfortably explore, but no more than 10 min. Gentle nudges were given when needed for all mice to cross the tube. On study days 10, 11, and 12, each cage underwent a round of tube testing based on previous methods (49). Each cage competed in three total rounds of tube testing. Mice from each cage competed in pairwise trials, with one mouse starting at each end of the tube. Upon entering, a timer was set for 2 min. Trials ended when the first mouse backed out of the tube and placed both hindfeet on the holding area floor. If no winner emerged by the end of 2 min, then it was considered a loss for both mice. Each pairing was replicated four times, yielding 40 total trials in cages of five mice and 12 trials in cages of three mice per round. The arena was cleaned with ethanol and allowed to dry between trials. Each mouse received a dominance score based on the number of trials won out of the number competed.

### Preputial Glands

On study day 13, mice were euthanized by prolonged exposure to CO_2_. Preputial glands were isolated, cleaned of connective tissue, and weighed in mg using an analytical balance (Ohaus, Parsippany, NJ). Each mouse's body length (tip of nose to base of tail) was also recorded in mm using calipers to calculate the preputial gland to body length ratio.

### PALS Score

The pelt aggression lesion scale (PALS) (27) was used to evaluate the final amount of wounding on each mouse. Currently PALS has only been validated to distinguish aggression-related wounding from ulcerative dermatitis and has not been directly linked to behavior. Additionally, while PALS is able to detect the presence of burst pigment follicles in black mice due to previous fighting (27), white mice do not possess this pigment. The ability to assess aggression history using PALS may be limited in white mice and will be explored in this study.

After preputial gland removal, pelts were removed from the carcass through gentle manipulation. The limbs were stretched and pinned so the pelt formed a rectangle and a subcutis image was taken of each pelt (Sony, Tokyo, Japan). A 9 × 9 grid was placed over each image and stretched from base of neck to base of tail. Each grid space was scored in terms of % visible area impacted and wound severity. Wound severity was assessed on a 0–4 scale with the following descriptions: (0) no visible damage; (1) five or fewer bites (double puncture sites); (2) more than five bite wounds with non-coalescing discoloration OR coalescing discoloration on <25% of the square; (3) coalescing discoloration on at least 25% of the square OR full thickness wounding covering <25% of the square; (4) full thickness wounding covering more than 25% of the square. Each grid space was given a score based on the following equation (27):

PALSgridScore = SeverityScore x AreaScore × 0.25.

Anterior, mid, and posterior regions were given an average score based on the three grid scores in each region. All analyses were done using the average posterior scores for each mouse, as it is most predictive of aggression related wounding (27).

### Statistics

#### Analysis Note

*N* = 24 cages were set up, but four cages of albino B6 (one group of five and three groups of three) were prematurely euthanized due to extreme aggression (Supplementary Table 1). Behavior data on day 2 were collected from one cage of albino B6-3 before euthanasia, and were included in SNA models. Additionally, a cage of SJL, group of three, was excluded due to dehydration from a faulty water sipper. Day 2 data from an SJL cage of five could not be observed due to camera malfunction. In total, there were *N* = 20 cages for SNA analyses and 19 for measure validation. Based on Mead's equation and the law of diminishing returns (40, 50), this sample size was large enough for sufficient error degrees of freedom in cage level models. Supplementary Table 2 provides details of experimental units used in each model described below.

#### Aggression Network Analysis

Analyses of aggression (referred to as aggression network analysis) were conducted based on previous methods for SNA. Occurrences of mediated and escalated aggression were combined into directed frequency sociomatrices for each cage (51). Each row and column corresponds to each individual within a cage, with actor and recipient mice represented by matrix rows and columns, respectively. Each value within a matrix tallies the number of times each “actor i” won an attack over each “recipient j.” In this study, all observed contests were won by the mouse who initiated them, so these values represent both the number of fights initiated and fights won. For each contest, the first mouse who fled or performed a submissive upright posture was considered the loser. Directed binary sociomatrices were also calculated from each cage's frequency matrix to yield presence/absence data. This indicates whether each “actor i” was ever observed attacking each “recipient j.”

The following global hierarchy measures were calculated using data from the binary sociomatrices: **Density**- the proportion of all possible interactions that occurred within a cage (51); **Directional Consistency (DC)**- a proportion of interactions that occurred from the most frequent direction to the least frequent direction within each dyad. DC scores closer to one indicate unidirectional interactions and scores closer to 0 indicate interactions that are more equally reciprocated. A measure of hierarchy linearity was not done as the interactions in this dataset were so skewed in favor of the alpha male that ranks between other cage mates were not stable enough to calculate a measure such as Landau's H or triangle transitivity (data not shown).

Individual social hierarchy ranking was calculated from the frequency sociomatrices using the **Glicko Rating System** (52). In brief, individuals lose points for every social defeat and win points for every victory. However, the number of points won/lost is dependent on the score difference between the opponents. E.g., if an actor defeats a recipient that has a much lower rating than itself, the actor will receive fewer points than if defeating a recipient with a rating that is close to its own. Rating certainty is also calculated based on the number of contests each individual engages in and the time since the last contest. There is more rank certainty in individuals who compete more frequently. For further explanation and evaluation of the Glicko System, please refer to (9). Since Glicko ratings have a default value of 2200, the net change in score was calculated for each mouse in order to better account for the variation in interaction frequency between cages and for scores to be more intuitive (i.e., victim mice have negative scores). Additionally, **individual out-strength** (the number of times the individual performed a behavior) and **in-strength** (the number of times the individual was the recipient of a behavior) were calculated for each animal for aggression, submission, allo-grooming, and social investigation (53). All hierarchy and SNA measures were organized and calculated using R Studio (version 3.6.1) with the following packages: compete (54), sna (55), and PlayerRatings (56).

General linear models (GLM), general linear mixed models (GLMM), or generalized linear mixed models (GLIMM) were used to address the following aims:

(1) examine how power is distributed in the cage based on aggression density and DC (GLM).
(2) assess how individual change in Glicko score relates to time budget, proportion of time observed in proximity to a cage mate, and allo-grooming in- and out-strength (GLMM). Time budget data were condensed using Principal Component Analysis (PCA). Only components with eigenvalues over 1.0 were analyzed in a GLMM.
(3) Evaluate the relationship between aggression in-strength and submission out-strength (i.e., do attack victims respond appropriately with submission; GLMM); and
(4) explore how displays of social investigation correlate with those of aggression and how likely recipients are to respond with submission (GLIMM).

All model assumptions were checked *post-hoc* and transformations were made when needed. In all models, strain, group size, and the interaction are included as fixed effects. All data were originally analyzed with day and all 2- and 3- way interactions as fixed effects. Non-significant interactions were dropped from all models. If day was not a significant factor, data were summarized for the study week and reanalyzed. Since each cage only contained one strain and group size, each factor was nested within cage and included as random effects for models addressing aims 2–4. It was also included in aim 1 models that included day as a fixed effect. For models addressing aims 2–4, mouse nested within cage was included as a random effect if the model tested the effect of day. Models for aims 1–3 were run in JMP Pro (version 14.0.0) with *post-hoc* Tukey tests where applicable. For aim four, correlation was assessed in JMP Pro using Pearson's correlation coefficient and a logistic regression model of each social investigation occurrence was run in SAS using PROC GENMOD with Bonferroni corrected *post-hoc* contrasts (alpha = 0.05/6 comparisons = *P* < 0.0083). An occurrence was given a 1 if submission occurred within 5 s of the social investigation, otherwise it was assigned a 0. For aim 4, only cages that had behavior observations from both study days were analyzed (Supplementary Table 2). Data organization and filtering for aim 4 were done in R studio using the tidyverse package. All figures were made in R Studio using ggplot2 and cowplot packages.

#### Dominance Measure Validation

Dominant and subordinate mice from each cage were determined using the change in Glicko score from interactions over both days of video. However, since the subordinate's behavior can be more indicative of dominance than aggression (3), Glicko scores were recalculated for each cage to reflect submissive behavior (Glick-Sub). These scores reflect all submission performed in response aggression, social investigation, or approach behavior. The mice in each cage with the highest and lowest Glicko-Sub scores were considered the respective dominant (mice who received the most submissions) and subordinate (mice who received the least submissions). The original scores from the aggression network analysis (Glicko-Agg), which specifically distinguishes aggressor from victim mice, were compared to other measures of focus to determine if they reflect both dominance and aggression. Darcin, fecal boli count in OFM, proportion of time in the center of the OFM, scores from three rounds of tube tests, PALS, and preputial data were only analyzed from these designated mice (38 total). However, two mice did not produce urine, causing missing values for darcin, and were excluded from the convergent validity factor analysis (*N* = 36 mice; Supplementary Tables 1, 2).

An exploratory factor analysis was done to determine if darcin, scores from three rounds of tube tests, PALS, and preputial data have convergent validity with the net change in both Glicko scores. First, all measures were standardized and run in GLMMs to isolate effects from strain and group size. Using previous methods (57), the residuals from the darcin, tube test, PALS, and preputial models were run in a factor analysis of correlations using JMP Pro. Maximum likelihood was used as the factoring method and prior communality was based on the squared multiple correlations. Varimax rotation was used on the loadings to improve factor interpretation. Loading threshold was set at 0.45 as done previously, since it is a mid-range value between what is used by behaviorists and biostatisticians (57). This analysis maintained the 5:1 subject to variable ratio for factor analysis (36 subjects/6 variables = 6). Scores from the resulting factors were tested in GLMs for direct effects on the change in Glicko-Sub and Glicko-Agg.

To assess discriminant validity, first scores from three rounds of tube tests were condensed in a principal component analysis. Only the first axis had an eigenvalue over one and represented all three scores (loading values over 0.90; Supplementary Table 3). The scores from this axis (tube test PC) were analyzed using a GLMM to test effects of fecal boli count in OFM, proportion of time in the center of the OFM, total distanced moved in the OFM, strain, and group size. Batch number and time of testing were included as blocking factors, but neither factor was significant, so they were dropped from the final model. Cage nested within strain and group size was included as a random effect.

## Results

### Aggression Network Analysis

#### Aim 1: How Strain and Group Size Affect Power Distribution

**Figure 1:**
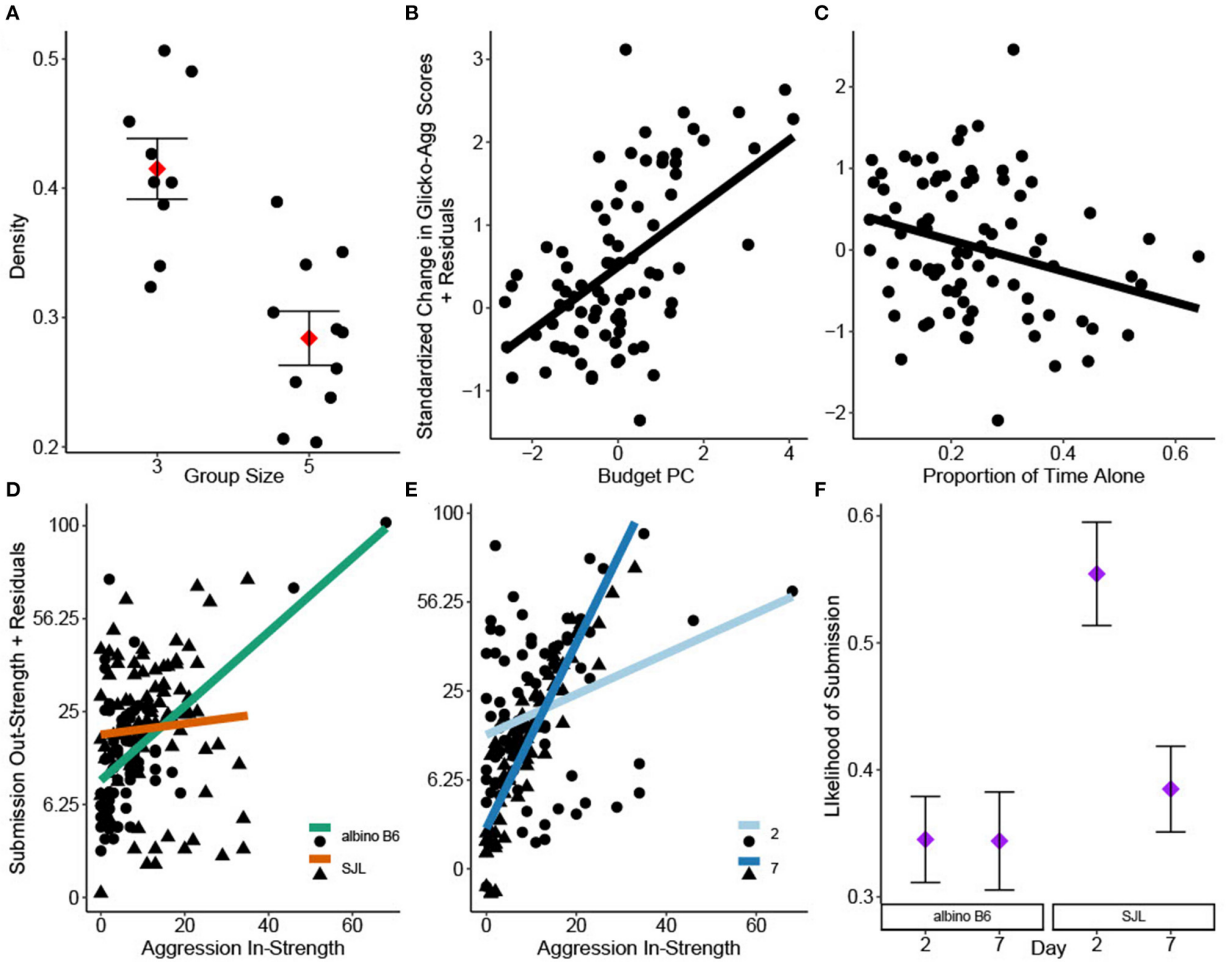
Social network analyses of group housed albino B6 and SJL male mice. **(A)** Group size significantly impacted aggression density (adj. R^2^ = 0.57, *N* = 20). Change in individual Glicko-Agg score was impacted by **(B)** Budget PC1 and **(C)** the proportion of time observed alone (adj. R^2^ = 0.12, *N* = 82). Interactions of **(D)** strain*aggression in-strength and **(E)** day*aggression in-strength significantly influenced individual submission out-strength (adj. R^2^ = 0.39, *N* = 156). Y axes in D and E are shown on a square root back transformed scale. **(F)** Binary logistic regression revealed a significant strain*day interaction on the likelihood that social investigation is followed by submission (*N* = 2192). Data in A and F are presented as factor level LSM ± SE. Data in A are presented over the scatter of individual residual points.

Aggression density was only significantly impacted by group size [GLM: *F*_(1, 14)_ = 17.43, $\eta_{\text{p}}^{2}$ = 0.55, *P* < 0.001], with cages of three showing higher density than cages of five (Figure 1A). However, all density values were low [Inter quartile range (IQR): 0.26–0.48]. Aggression DC was not significantly impacted by strain or group size [GLM strain: *F*_(1, 14)_ = 0.17, *P* = 0.69; group size: *F*_(1, 14)_ =0.22, *P* =0.65] and was generally high across cages (IQR: 0.72–0.90).

#### Aim 2: Influence of Time Budget, Cage Mate Proximity, and Allo-Grooming on Glicko-Agg Score

PCA of time budget data yielded one significant component (Budget PC), with all behaviors loading strongly. Time spent active and performing solitary sleep had high positive loadings while time spent in group sleep had a high negative loading (Table 2). Scores from Budget PC had a positive relationship with the change in Glicko-Agg score [GLMM: *F*_(1, 69.15)_ = 24.46, $\eta_{\text{p}}^{2}$ = 0.26, *P* < 0.001; Figure 1B] while the proportion of time observed alone, based on location data, had a negative relationship [GLMM: *F*_(1, 72.39)_ = 5.02, $\eta_{\text{p}}^{2}$ = 0.06, *P* = 0.028; Figure 1C]. As time alone increased, the change in Glicko-Agg scores decreased. Neither allo-grooming in-strength nor out-strength had a significant effect on the change in Glicko-Agg score [GLMM: *F*_(1, 45.03)_ = 1.12, *P* = 0.296; *F*_(1, 71.23)_ = 0.81, *P* = 0.371].

**Table 2:** Loading values from Principal Component Analysis of time budget behaviors.

	**Budget PC**
Solitary sleep	0.75601
Group sleep	−0.97370
Active	0.69162
Eigenvalue	2.00
Total variance explained (%)	66.60

*Only the first component was interpreted based on eigenvalue analysis. Scores from this component were used in a GLMM*.

#### Aim 3: Relationship Between Submission Out-strength and Aggression In-strength

Submission out-strength was significantly impacted by the strain*aggression in-strength interaction [GLMM: *F*_(1, 120)_ = 7.21, $\eta_{\text{p}}^{2}$ = 0.06, *P* < 0.001] as well as the day*aggression in-strength interaction [GLMM: *F*_(1, 124.1)_ = 34.83, $\eta_{\text{p}}^{2}$ = 0.22, *P* < 0.001]: albino B6 mice and mice on day 7 performed more submissions relative to the attacks they received (Figures 1D,E).

#### Aim 4: How Social Investigation Relates to Aggression and Submission

There was a high correlation between social investigation out-strength and aggression out-strength (Pearson's R = 0.79, *P* < 0.001, 95% CI: 0.72–0.84). Logistic regression was used to assess the likelihood of submission occurring within 5 s of a social investigation. There was a significant strain*day interaction on this likelihood (GLIMM: $\chi_{1}^{2}$ = 5.76, *P* = 0.016). The probability of submission after social investigation was highest in SJL mice on study day 2 (Tukey: *P* < 0.002, Figure 1F).

### Dominance Measure Validation

Strain had a significant effect on the following dominance measures: preputial gland ratio [GLMM: *F*_(1, 13)_ = 9.17, η^2^= 0.41, *P* = 0.009]; darcin [GLMM: *F*_(1, 12)_ = 55.53, η^2^= 0.82, *P* < 0.001]; and PALS score [GLMM: *F*_(1, 13)_ = 38.58, η^2^= 0.75, *P* < 0.001]. Preputial ratios and darcin levels were higher in albino B6 mice while PALS scores were higher in SJL mice. A strain * group size interaction impacted tube test scores from round 1 [GLMM: *F*_(1, 13)_ = 7.12, *P* = 0.019], but *post-hoc* Tukey tests showed no significant differences. Further, strain impacted Glicko-Sub score [GLMM: *F*_(1, 13)_ = 5.45, η^2^= 0.30, *P* = 0.036]. Please refer to Supplementary for strain*group size least square means. The random factor, CageID, was significant in PALS (*P* = 0.029), Glicko-Sub (*P* = 0.003), and Glicko-Agg (*P* = 0.002) models. Correlation values for all variables are presented in Supplementary Table 5. Notably, Glicko-Agg and Glicko-Sub scores were highly correlated (Pearson's *R* = 0.97, *P* < 0.001, 95% CI: 0.95–0.99).

**Figure 2:**
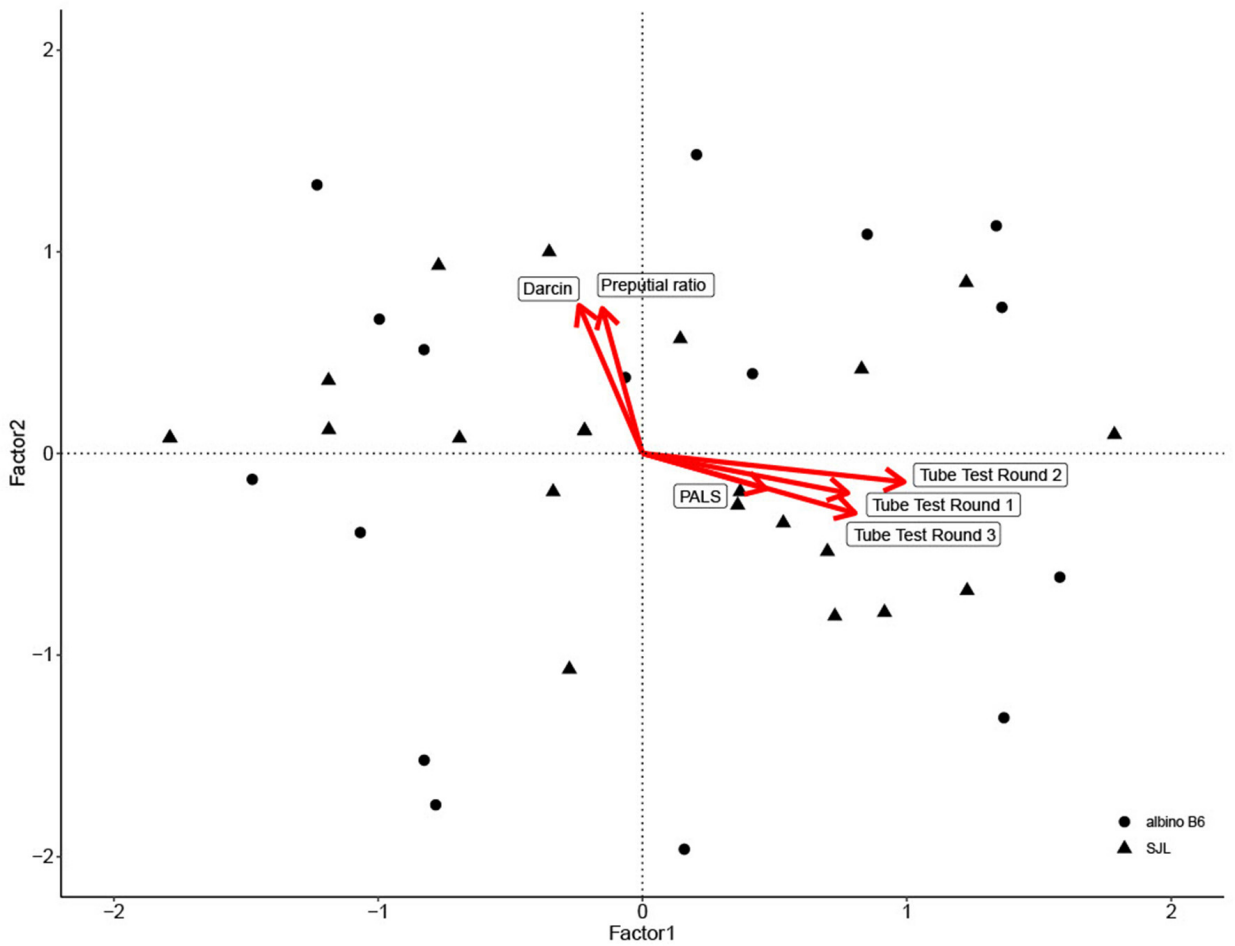
Biplot of factor analysis used to assess convergent validity. Individual data point scores are plotted along Factor 1 and Factor 2, with shape based on strain. Variable loadings for each factor are depicted by red arrows.

For convergent validity, eigenvalue analysis showed that two factors were sufficient to interpret the dataset. The first factor accounted for over 42% of total variation and reflected scores from all three rounds of tube testing and PALS score. The second factor accounted for over 20% of total variation and reflected urinary darcin, and preputial gland ratio (Table 3; Figure 2). Factor two was a significant predictor of both Glicko-Sub score [GLM: *F*_(1, 35)_ = 15.70, η^2^= 0.31, *P* < 0.001] and Glicko-Agg score [GLM: *F*_(1, 35)_ = 20.86, η^2^= 0.37, *P* < 0.001].

**Table 3:** Loading values from factor analysis to assess convergent validity of measure residuals.

	**Factor 1**	**Factor 2**
Preputial gland: body length ratio	−0.152076	0.719176
Urinary darcin	−0.239981	0.734438
Average posterior PALS score	0.467036	−0.176451
Tube test- round 1	0.777146	−0.197103
Tube test- round 2	0.989930	−0.141560
Tube test- round 3	0.804816	−0.296798
Eigenvalue	2.53	1.23
Total variance explained (%)	42.18	20.58

*Values below the loading threshold of 0.45 are presented in gray. Factors with eigenvalues over 1 are shown*.

For discriminant validity, neither the number of fecal boli [GLMM: *F*_(1, 25.93)_ = 0.80, *P* = 0.381], proportion of time in the center of the OFM [GLMM: *F*_(1, 19.34)_ = 0.04, *P* = 0.851], nor total distance traveled [GLMM: *F*_(1, 15.42)_ = 1.82, *P* = 0.196] were significant predictors of the tube test PC.

## Discussion

The main aim of this study was to assess the convergent validity of dominance measures with home cage rankings based on SNA in group housed male mice. Additionally, the discriminant validity of the tube test was assessed in relation to measures of anxiety and locomotion from the OFM. Although dominance in some situations is best predicted by subordinate behavior instead of aggressive behavior (3), Glicko scores in our study calculated from both aggression and submission data were highly correlated. For the cages used in this study, aggression was likely a good indicator of dominance. Additionally, both scores were predicted by the same factor representing urinary darcin and preputial gland: body length ratio. This suggests that both measures show convergent validity with home cage behavior. This extends the patterns found in previous work on males reared in isolation or tested in complex competition arenas (28, 29, 32–34). The correlation between preputial gland ratio, urinary darcin, and home cage aggression is likely testosterone mediated since all three are testosterone dependent (32, 58, 59). In fact, testosterone treated females are more aggressive and their urine can trigger intense attacks toward castrated males and normal females, which supports an olfactory based mechanism behind aggression (60, 61). However, it is likely dependent on more complex, tissue specific levels of testosterone and receptor density since circulating levels have been shown to not predict individual wounding or aggression levels (62). This solidifies the utility of the preputial gland ratio and urinary darcin to indicate individual dominance ranking, keeping in mind that both measures were strain dependent. As shown here and previously, mice of the C57 lineage produce more darcin than mice of Castle or Swiss lineages (63–65). Further, albino B6 mice also had larger preputial gland ratios than SJL mice. To the best of our knowledge, strain effects on gland ratio have not been previously examined. However, based on the η^2^ for each model, strain had a stronger effect on darcin than preputial gland ratio. This may explain why darcin accounted for so much less variability in Glicko score than preputial ratio.

On the other hand, the average posterior PALS score loaded on a factor that did not predict either aggression-based or submission-based Glicko score. As discussed below, the cages here primarily displayed despotic hierarchies, but the level of wounding varied across cages. For cages that display aggression with more forceful biting, the level of wounding could be a powerful indicator of dominance. However, it will not be as predictive for cages that primarily display mediated forms of aggression like mounting and chasing and it would not predict social rank based on submission in interactions that do not involve physical contact. Additionally, the relationship may not be as clear for more linear relationships where there is conflict between mid-ranking mice. Since this is the first direct comparison between PALS score and behavior, further work will have to examine its value in different social structures. Additionally, this relationship could have been impacted by the mice's pigmentation. PALS' predictive ability has been validated in black mice where burst pigment follicles indicate mild aggression (27). Since the white mice used here do not have these follicles, only more severe wounding could be documented. This limits PALS' predictive ability in cages of white mice that display more mediated aggression. However, these findings support the robustness of using darcin and preputial gland ratio as they correspond with dominance behavior, regardless of how much vascular damage may be present.

Scores across three rounds of tube tests also loaded on an axis that did not predict Glicko score. This result was surprising since previous work has found tube test rankings to correlate with agonistic behavior both in the home cage and in an unfamiliar setting (26, 49), so we expected that tube test scores would at least predict Glicko-Agg scores. In one case, the difference could be due to previous assurance of rank stability in the tube test, which was not done here (26). Additionally, these data could be a product of their respective environments: aggressive behavior used to calculate Glicko scores was recorded in the home cage while tube test scores were from a specialized arena. It is possible that the relationship here reflects the tendency for some subordinate mice to regain confidence when away from their attacker (11). However, it has also been shown that many hierarchies based on the tube test produce unclear ranks over time, which could indicate that dominance ranks have a transient nature (19), or it could reflect another trait all together. The data reported here support the latter option since scores from all rounds showed high correlation and loaded on the same factor. Interestingly, posterior PALS score had a weak relationship with the same factor as tube test scores. This is the first known comparison between tube test scores and wounding, but since most aggression related wounding is located in a posterior region, it would be advantageous for mice who are already injured to remain in the tube to prevent further attacks from behind. In relation to OFM measures, tube test scores displayed good discriminant validity, implying that general locomotion and anxiety in a novel environment do not influence tube test performance. The lack of relationship with distance moved confirms past work (26). In terms of anxiety, mice experienced the OFM and acclimation to the tube test arena before testing, so it is possible that they displayed less anxiety each time they left the cage. Interestingly, in both factor analyses, scores from all three tube test rounds loaded strongly on the same factor. Previously, it has been shown that tube test scores are more consistent between the second and third round, suggesting that mice must be repeatedly tested for stable scores (18, 66). These conflicting results may be reflective of strain or environmental conditions: the former studies used pigmented C57BL/6 mice tested in facilities outside the Unites States. Facility to facility environmental differences are known to influence behavioral data across several strains of mice (67).

Taken together, measuring urinary darcin or the preputial gland: body length ratio would be a more practical alternative for researchers than time intensive home cage observations. However, both measures have their draw backs: preputial gland ratio comes with the challenge of being an end of life measure while darcin is more impacted by strain variation. If it is feasible to only determine social rank at the end of the study, then preputial ratio is suggested. Otherwise, urinary darcin may be more advantageous depending on strain.

This project also aimed to better understand how individual aggression patterns relate to other home cage behaviors through aggression focused SNA. While previous SNA work has provided valuable insight on mouse social dynamics, it was either based on limited, live person sampling that may only reflect behavior at certain times or used large vivarium housing that may not accurately represent the conditions most laboratory mice experience in a typical shoebox cage.

For group level measures, our data revealed that aggression density is primarily low, and DC is high in these two strains of male laboratory mice. This indicates that key mice within each cage consistently perform aggression and the attacks are not typically reciprocated. This matches previous work which found that male mice often display despotic power structures (5, 6, 8, 10). Past reports show that linear hierarchies are also common (5–7, 9–12), however the interactions in this dataset were so skewed in favor of the alpha male that ranks between other cage mates were not stable enough to calculate a traditional linearity measure such as Landau's H or triangle transitivity (data not shown). The only significant treatment effect in this experiment indicates that group size influenced aggression density: cages of three had higher density than cages of five. Although data were analyzed as a proportion in order to account for more mice and potential interactions in groups of five, this difference may still be due to the fact that fewer mice in a cage inherently reduces the number of potential interactions, so a single pair-wise interaction will have a larger impact on density.

In terms of the individual, Glicko-Agg scores were only impacted by time budget, as represented by the Budget PC and the proportion of time observed alone in the cage. PCA of time budget behaviors (active, group sleep, and solitary sleep) revealed that mice who were more active spent more time sleeping alone. These same mice who were more active and performed more solitary sleep, had a higher change in Glicko-Agg score over the study week. To the best of our knowledge, how aggression relates to activity in the home cage has not been formally studied, but this pattern is consistent with previous work using a resident intruder paradigm. Mice that undergo social defeat daily and then are housed separately from their attacker, using a cage partition, show reduced activity, and display characteristics of depression (68–70). However, this could also represent a higher motivation to patrol territory in dominant, aggressor mice, who are known to claim territory through scent marks more than subordinates (71). These results also confirm anecdotal observations and past work that more aggressive mice rest away from cage mates (5). However, this contrasts with the negative relationship seen between the proportion of time observed alone and the change in Glicko-Agg score. This is likely because the time observed alone accounts for both active and inactive periods. Mice who are frequently targeted by an aggressor have been shown to actively avoid them, particularly when there is a despotic dynamic (7), so it is possible that the pattern seen here is representative of active times when subordinate mice are fleeing from their aggressor. Additionally, the amount of allo-grooming performed and received by these inbred strains did not relate to the change in Glicko score, which agrees with past work on outbred mice showing little correlation between position in a grooming network and the position of individuals in networks derived from other behaviors (9). However, this does not necessarily mean that allo-grooming is solely motivated by affiliation in laboratory mice. If it did, we would expect a negative relationship between change in Glicko score and the amount of grooming performed. It has been suggested that allo-grooming may serve a dual purpose by providing emotional support between subordinate mice and acting as reconciliation when done by dominants after aggression (9). The latter has been frequently observed in primates (72–74); however assessing the direct sequence pattern of allo-grooming was beyond the scope of this study and would be a worthwhile future topic.

The Glicko-Agg score model, and those mentioned above for density and DC, used combined data from the two days over the course of the study week, since study day did not have an impact on these measures. This suggests that, in albino B6 and SJL mice, dominant males emerge by the end of the second housing day and mice maintain their social rank, at least over the first week of housing. Previous work with CD-1 mice showed a similar pattern, however a subset of those observed groups took over 2 weeks to stabilize ranks, which may be a product of that strain (6).

In general, the amount of submission the mice performed was positively related to the number of attacks they received, aligning with past work on outbred mice (6). The interaction of day* aggression in-strength showed that submission rate was best explained by aggression on study day 7. Additionally, the interaction of strain* aggression in-strength showed that albino B6 mice performed more submission in relation to the number of times they were attacked. In fact, the fitted line relating aggression in-strength and submission out-strength for SJL mice only has a slightly positive slope, implying that this relationship was primarily seen in albino B6 mice. However, this is not to suggest that SJL mice do not submit when attacked, only that their submission rate cannot solely be explained by attacks. The likelihood that a mouse would submit after social investigation was higher for SJL mice on study day 2, which likely impacted the relationship depicted by both interactions in this model. One point of consideration is that SJL mice had higher PALS scores than albino B6, so even though the number of attacks did not vary across days, those from SJL mice presumably caused more physical damage. However, a downfall of PALS is that it is a cumulative, end of life measure, so it cannot differentiate between a recent, gentler attack and one that was more severe and partially healed. Still, it is likely that more damage was caused by attacks at the start of the study since male mice are less tolerant of each other when they are unfamiliar (35, 75). This may have triggered subordinate SJLs to perform more submission on day 2 in response to sniffing to prevent the interaction from escalating into an attack, since there was high correlation between the number of times an individual attacked and sniffed a cage mate. This high correlation confirms previous work (9, 21, 76) and suggests that the motivation for social investigation may not always be neutral, as previously considered (37–39).

## Conclusion

In summary, this study showed that urinary darcin and preputial gland: body length ratio have good convergent validity with home cage aggression, both mediated and escalated behaviors, and would be a practical alternative to home cage observations for identifying individual dominance rank. However, both are subject to strain variation and preputial ratio must be done as an end of life measure. Additionally, tube test scores have good discriminant validity with measures of locomotion and anxiety from the OFM. Finally, these data confirm that despotic power structures are prevalent in male social groups of inbred laboratory mice, aggressors are often more active and rest away from other cage mates, and that social investigation behaviors can be linked to aggression. This information provides more understanding of mouse home cage behavior and can be utilized to help develop aggression mitigation strategies.

## Data Availability Statement

All raw behavior data and scripts from SNA are published as Supplementary Material. Raw urinary protein files are available from the Mass Spectrometry Interactive Virtual Environment (MassIVE) repository (file ID: MSV000086740).

## Ethics Statement

The animal study was reviewed and approved by Purdue University Animal Care and Use Committee.

## Author Contributions

AB led sample collection, video coding, and wrote the manuscript. AB and BG performed data analyses. All authors designed and planned this study and contributed to the article and approved the submitted version.

## Conflict of Interest

The authors declare that the research was conducted in the absence of any commercial or financial relationships that could be construed as a potential conflict of interest.

## Supplementary Material

Video 1 Tail rattle example.

Video 2 Escalated aggression example.

Video 3 Allo-groom + mount example.

Data Sheet 1 Raw behavior data.

Data Sheet 2 Supplementary Tables and Figures.
