# Supplementary material for: Who's the Boss? Assessing Convergent Validity of Aggression Based Dominance Measures in Male Laboratory Mice, Mus Musculus
Source: Front Vet Sci. 2021 Jul 9;8:695948. doi: 10.3389/fvets.2021.695948 (PMC8301077; doi:10.3389/fvets.2021.695948)
Supplement: Data Sheet 2 — Supplementary Tables and Figures. [file Data_Sheet_2.pdf]

Supplementary Material

1 Supplementary Figures and Tables

Supplementary Table 1. Count of cages used for final analysis, broken down by strain and group size.

	Total Cages	SJL- 3	SJL- 5	Albino B6- 3[1]	Albino B6- 5
Social network analysis	20	5	6	4	5
Dominance measure- convergent validity	18	4	6	3	5
Dominance measure- discriminant validity	19	5	6	3	5

[1]One cage was euthanized between day 2 and 7 of video data. Data from day 2 was included in social network analyses.

Supplementary Table 2. Count of experiment units (either cage or mouse) used in each social network analysis and dominance measure model. The number of video days observed is indicated where applicable.

	Cages	Mice	Sampling Unit	Units with two days of behavior data	Units with one day of behavior data
**Social network analysis**					
Aim 1- power distribution	20	82	cage	18	2
Aim 2- influences on Glicko score	20	82	mouse	74	8
Aim 3- relationship between submission performed and aggression received	20	82	mouse	74	8
Aim 4- likelihood of submission following social investigation	18	74	mouse	74	0
**Dominance measures**					
Glicko score	19	38	mouse	36	2
Preputial gland ratio	19	38	mouse	---	---
Time in center of OFM	19	38	mouse	---	---
Fecal boli in OFM	19	38	mouse	---	---
Darcin	18	36	mouse	---	---
Tube test scores	19	38	mouse	---	---
Average posterior PALS score	19	38	mouse	---	---

Supplementary Table 3. Loading values from principal component analysis of tube test scores over three rounds. Only the first component had an eigenvalue over 1.

	Tube Test PC
Tube Test Round 1	0.90062
Tube Test Round 2	0.96658
Tube Test Round 3	0.90369
**Eigenvalue**	2.56
**Total variance explained (%)**	85.4

Supplementary Table 4. Least square mean ± SE for each strain*group size combination from general linear models of standardized measures tested for convergent validity.

	SJL – 3	SJL – 5	Albino B6 - 3	Albino B6 - 5
Change in Glicko-Agg score	-0.0101 ± 0.0616	-0.0068 ± 0.0601	0.1098 ± 0.0760	0.0747 ± 0.0685
Change in Glicko-Sub score	-0.0566 ± 0.0847	-0.0520 ± 0.0827	0.2011 ± 0.1046	0.0404 ± 0.0942
Preputial gland ratio	-0.2622 ± 0.3138	-0.3545 ± 0.3065	0.4057 ± 0.3876	0.6597 ± 0.3489
Urinary darcin	-0.5546 ± 0.2374	-0.6397 ± 0.2250	0.7949 ± 0.2836	1.1178 ± 0.2565
Tube test score-round 1	0.1077± 0.2221	-0.5201 ± 0.2169	-0.6075 ± 0.2743	-0.1921 ± 0.2470
Tube test score-round 2	0.2318 ± 0.2421	-0.3185 ± 0.2364	-0.4560 ± 0.2990	-0.1167 ± 0.2692
Tube test score-round 3	0.0885 ± 0.2778	-0.1854 ± 0.2713	-0.5723 ± 0.3431	-0.1225 ± 0.3089
Average posterior PALS score	0.6155 ± 0.2037	0.6368 ± 0.1989	-0.5242 ± 0.2515	-0.4631 ± 0.2265

Supplementary Table 5. Correlation coefficients of dominance measure residuals used in the factor analyses and subsequent general linear models.

	**Change in Glicko-Agg score**	**Change in Glicko-Sub score**	**Preputial gland ratio**	**Urinary darcin**	**Tube test score-round 1**	**Tube test score-round 2**	**Tube test score-round 3**	**Average posterior PALS score**
Change in Glicko-Agg score	1.0000	0.9762	0.6528	0.4514	-0.3280	-0.2747	-0.3071	-0.5291
Change in Glicko-Sub score	0.9762	1.0000	0.6209	0.4004	-0.3143	-0.2893	-0.2996	-0.5869
Preputial Gland Ratio	0.6528	0.6209	1.0000	0.5583	-0.2983	-0.2524	-0.2808	-0.4193
Urinary darcin	0.4514	0.4004	0.5583	1.0000	-0.3169	-0.3460	-0.4817	-0.0280
Tube test score-round 1	-0.3280	-0.3143	-0.2983	-0.3169	1.0000	0.7972	0.6274	0.4409
Tube test score-round 2	-0.2747	-0.2893	-0.2524	-0.3460	0.7972	1.0000	0.8387	0.4873
Tube test score-round 3	-0.3071	-0.2996	-0.2808	-0.4817	0.6274	0.8387	1.0000	0.4507
Average posterior PALS score	-0.5291	-0.5869	-0.4193	-0.0280	0.4409	0.4873	0.4507	1.0000

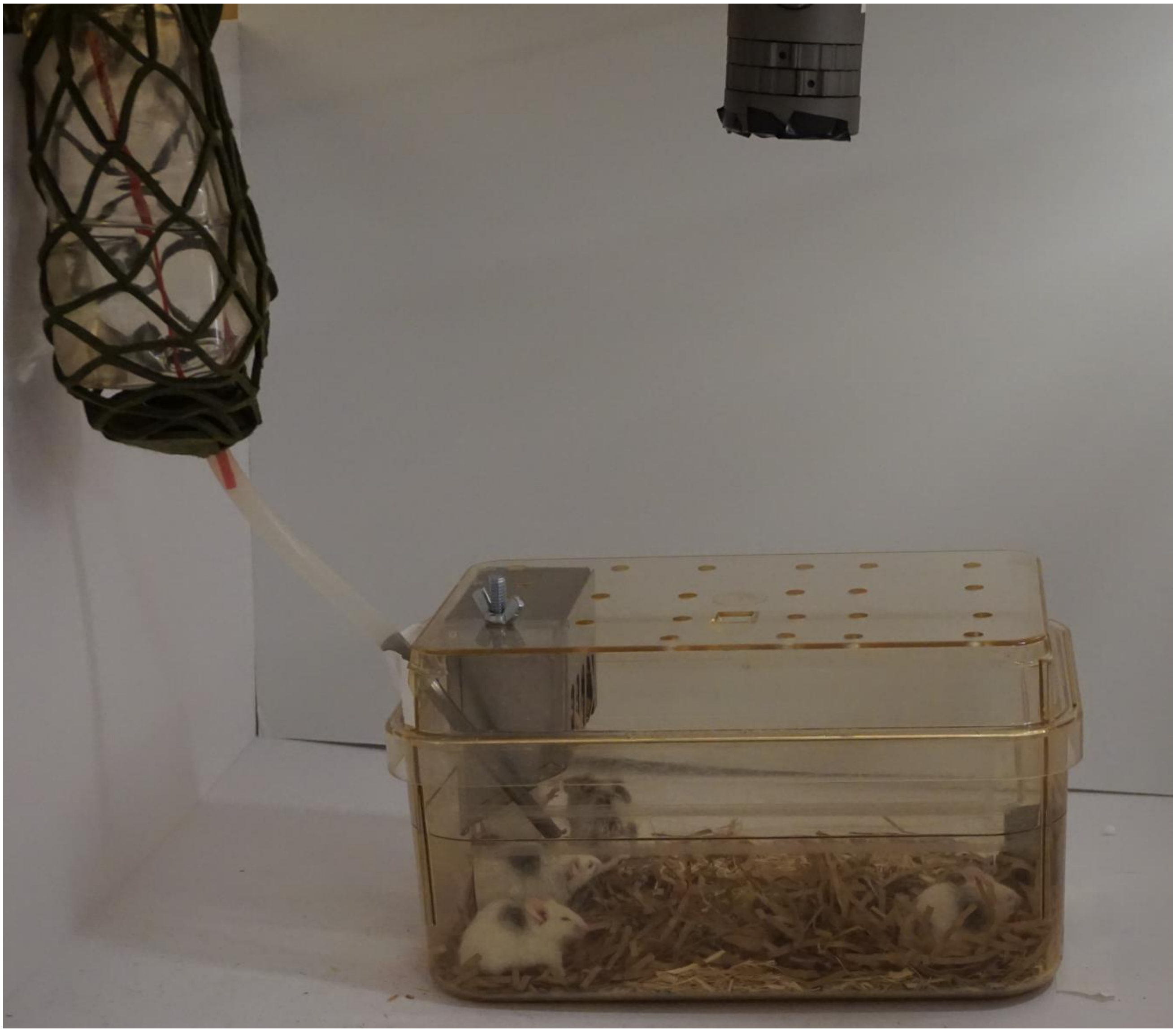

Supplementary Figure 1. Custom caging for home cage observations. Holes were drilled into polysulfone lids for air exchange on static racks. A metal feeder was secured to the lid using a nut and bolt. An external water bottle was accessible through a hole in the side of the lid and connected using medical grade silicone tubing and a metal water sipper. These cages allowed for overhead monitoring using CCTV cameras, one of which can be seen at the top of the figure.
